# Novel acoustic flat focusing based on the asymmetric response in parity-time-symmetric phononic crystals

**DOI:** 10.1038/s41598-019-46467-3

**Published:** 2019-07-11

**Authors:** Hang Yang, Xin Zhang, Yuechang Liu, Yuanwei Yao, Fugen Wu, Degang Zhao

**Affiliations:** 1 School of Physics and Optoelectronic Engineering, Guangdong University of Technology, Guangzhou, 510006 China; 2 School of Information Engineering, Guangdong University of Technology, Guangzhou, 510006 China; 3 School of Physics, Huazhong University of Science and Technology, Wuhan, 430074 China

**Keywords:** Acoustics, Condensed-matter physics

## Abstract

We present a two-dimensional (2D) parity-time-symmetric (*PT*-symmetry) phononic crystals (PCs) with balanced gain and loss medium. Using the super cell method of rectangular lattice, we exhibit the thresholdless spontaneous *PT*-symmetry breaking in the band structure. The numerical results show that the asymmetric scattering properties obviously occur in a non-Hermitian system. At two specific incident frequencies, unidirectional reflectionless and perfect transmission behaviors exist individually in opposite directions, which are accompanied by a phase transition of π. Based on the generalized Snell’s law, combining such a *PT*-symmetric medium, we design a novel metamaterial crystal for *PT*-symmetric acoustic flat focusing. Its focus frequency can also be modulated by the gain/loss parameter. The novel flat focusing based on the *PT*-symmetry that we propose opens a new door for high-dimensional applications of non-Hermitian metamaterials in acoustic wave manipulation.

## Introduction

Over the past two decades, there have been many studies on acoustic wave manipulations. The periodically structured PCs, metamaterials and metasurfaces are well-designed for applications such as acoustic waveguide^1,2^, acoustic focusing^3–9^, acoustic cloaking^10^, acoustic collimation^11,12^, and unidirectional transmission^13–16^. In particular, in flat focusing, PCs are implemented in many interesting manners, including negative refraction^4–6^ and metasurfaces^7–9^. However, most studies focused on Hermitian system, except a few works related to the complex domain^17^. Since the concept of *PT*-symmetry was introduced in Bender’s work^18–20^, it began to arouse much interest on research of non-Hermitian systems, which remain invariant under the combination of parity (*P*) and time reversal (*T*) operations. Operator *P* is defined as: $\hat{p}\to -\,\hat{p}$, $\hat{x}\to -\,\hat{x}$, while operator *T* is: $\hat{p}\to -\,\hat{p}$, $\hat{x}\to \hat{x}$ and $i\to -\,i$, which requires the non-Hermitian systems to satisfy the condition: $V(\,-\,x)={V}^{\ast }(x)$. This condition is different from previous works and results in the emergence of the conjugate complex modulus, which has been widely explored in theory for electronic and optical systems^21–32^, and has introduced many interesting features.

Recently, non-Hermitian acoustics have been introduced to the 1D structure. The properties of one-way propagation^33^ and bound states of the defect mode^34^ have been systematically investigated. These acoustic studies are only studied in 1D space. Very recently, Zhu *et al*. designed a curved *PT*-symmetric metamaterials crystal in 2D space, to achieve the unidirectional sound focusing effect in simulation and experiment^35^. However, until now, there has been no comprehensive analysis about the effect on the dispersion relation caused by *PT*-symmetry breaking in 2D acoustic systems. In addition, not confined to a curved structure, achieving the optional geometric manipulation of *PT*-symmetric metamaterials is a challenging problem.

In this letter, we introduce a 2D *PT*-symmetric PC with complex parameters. A complex bulk modulus in active practical systems can be uesd^36^, and non-Hermitian acoustic systems are also proven to be feasible in practice^37–40^. We analyze the degenerated band structure which experiences thresholdless *PT*-symmetry breaking. Moreover, we study the asymmetric scattering properties caused by *PT*-symmetry breaking. The unique unidirectional reflectionless and perfect transmission behaviors with a phase transition of π are shown in the following sections. Based on the phase characteristic, which is associated with the generalized Snell’s law^7,41^, we further design a *PT*-symmetric planar metamaterials lens for acoustic focusing, which opens a new degree of freedom of geometric shapes for acoustic wavefront engineering in non-Hermitian systems.

## Results

### Band structure

**Figure 1:**
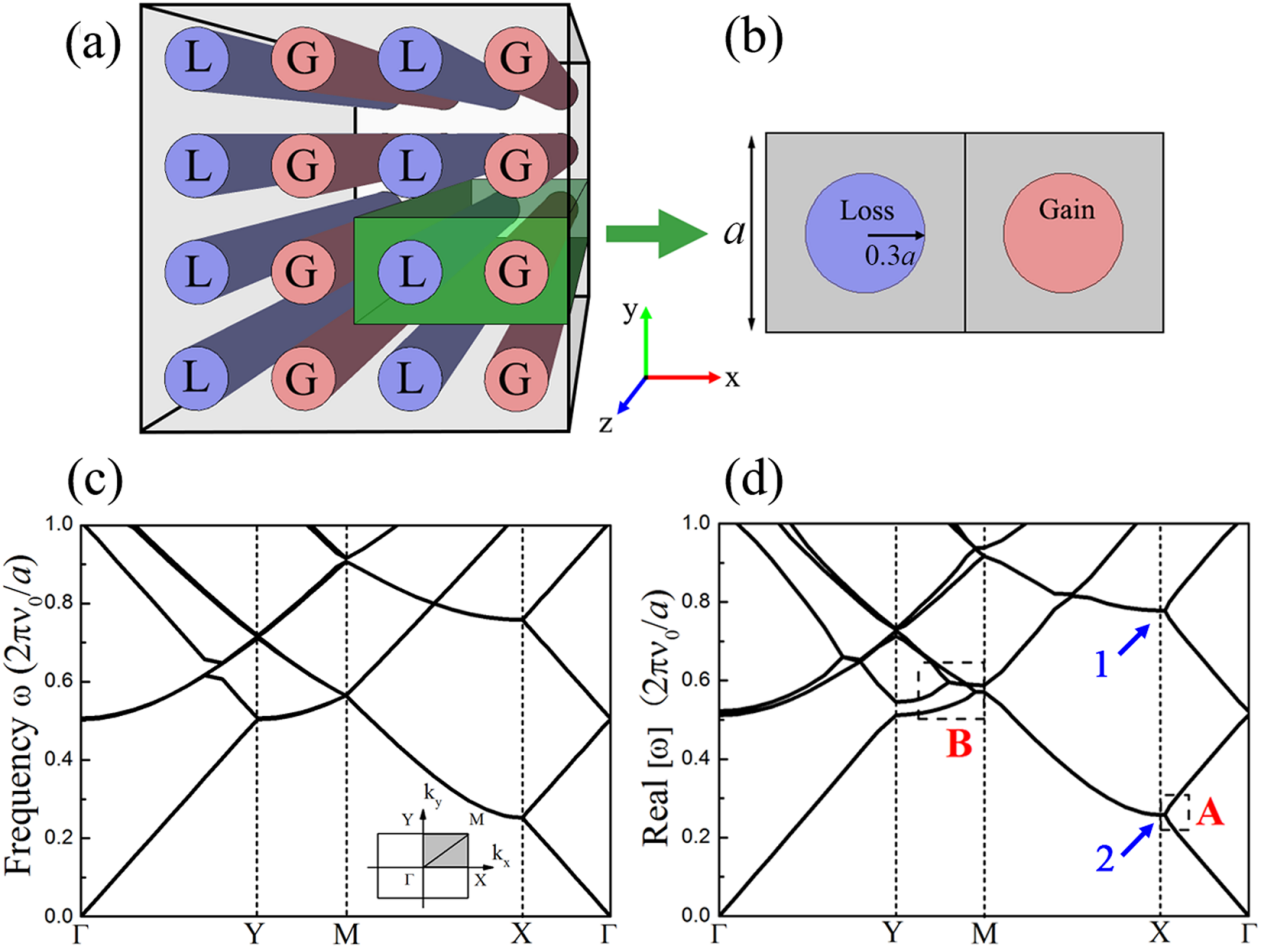
(**a**) Model of the 2D non-Hermitian systems (infinitely long in the z direction) composed of cylindrical rods embedded vertically in water. Rods labeled L and G represent the loss and gain regions respectively. (**b**) Plane schematic diagram of a 2 × 1 super cell in the structure. (**c**) Band structure of 2D Hermitian PCs for a rectangular lattice with *α* = 0. The first Brillouin zone of the rectangular lattice is indicated in the inset. (**d**) Real part of the band structure in the non-Hermitian system for a rectangular lattice with *α* = 0.25.

We propose a structure of 2D *PT*-symmetric PCs, which is presented in Fig. 1(a). The system is arranged alternately with two types of cylindrical rods with conjugate complex modulus, one of which is the gain, and the other is the loss. The longitudinal wave velocity of the gain (loss) materials has a positive (negative) imaginary part. Here, we use *α* to denote the imaginary strength of the gain/loss materials. The distribution in the x direction with balanced gain and loss satisfies the *PT*-symmetry condition that the real part of the modulus is an even function, while the imaginary part is odd. Figure 1(b) shows the diagram of the super cell marked as green in Fig. 1(a), which contains 2 × 1 unit cells with lattice constant *a* and a rod radius of 0.3*a*. The band structures of the underlying 2D Hermitian system and non-Hermitian system are calculated, respectively. Figure 1(c) shows the band structure of common PCs with only the real component (*α* = 0). The wave vectors sweep along the boundary of the irreducible Brillouin zone for rectangular lattice. Although the 2 × 1 super cell method was used, the calculation result is identical to that of the unit cell, except a band corresponding to the unit cell is folded into two bands in the x direction of the super cell. Some bands also cross and degenerate in the Brillouin zone. As shown in Fig. 1(d), thresholdless *PT*-symmetry breaking spontaneously occurs when the imaginary component (*α* = 0.25) is added to form a balanced complex elastic modulus. Many degenerated bands appear along the boundary, especially in the Γ-X and Y-M directions. Similar to the case for 1D structures, some bands merge at the high symmetry points (labeled as dashed box A). However, in dashed box B, the originally degenerated bands split and merge again with other multiple bands, which does not occur in low-dimensional situations. The compression of merged bands also causes the appearance of directional bandgaps.

**Figure 2:**
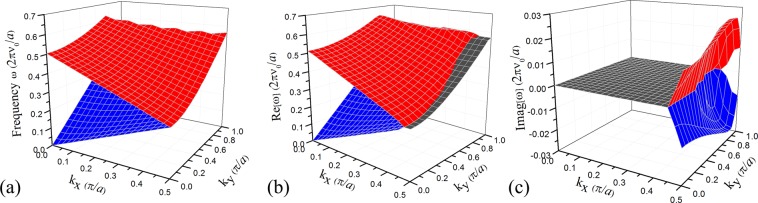
(**a**) Reduced frequencies for the first (blue) and second (red) super cell bands of the Hermitian system. (**b**,**c**) Real and imaginary parts of the frequencies for the first and second super cell bands, respectively. The dark gray regions indicate the merged bands.

To provide a vivid illustration of the degenerate phenomenon, the 3D dispersion surface for the first and second bands are plotted. The band structure for the super cell of the underlying Hermitian acoustic system (*α* = 0) is shown in Fig. 2(a). We observe a degenerate contour of frequency eigenvalues along the line of *k*_*x*_ = 0.5*π*/*a* in the band structure because of the fold features in the super cell system. When the gain and loss are added to form a non-Hermitian system with a coefficient *α* = 0.25, Fig. 2(b) shows that the degenerate contours at the band crossing instantaneously experience thresholdless spontaneous *PT*-symmetry breaking. A particularly interesting phenomenon occurs, where the folded bands merge together outwards from the primary degenerate contour, and a new contour appears at the boundary between the merged and the independent regions. Thus, degeneracy in 3D ***k***-space is exhibited as bands merging in a continuous area, which includes high-symmetry points and arbitrary low-symmetry points. Due to the extra spatial degree of freedom, the degenerate points in the 1D structure evolve into an irregular contour in the 2D system. A comparison of data values also shows that the bands nearly flatten in the x direction perpendicular to the degenerate contour after they merge. The imaginary part, as presented in Fig. 2(c), contains identical degenerate contour and forms complex conjugate pairs of frequencies with the real components.

### Properties of the asymmetric response

**Figure 3:**
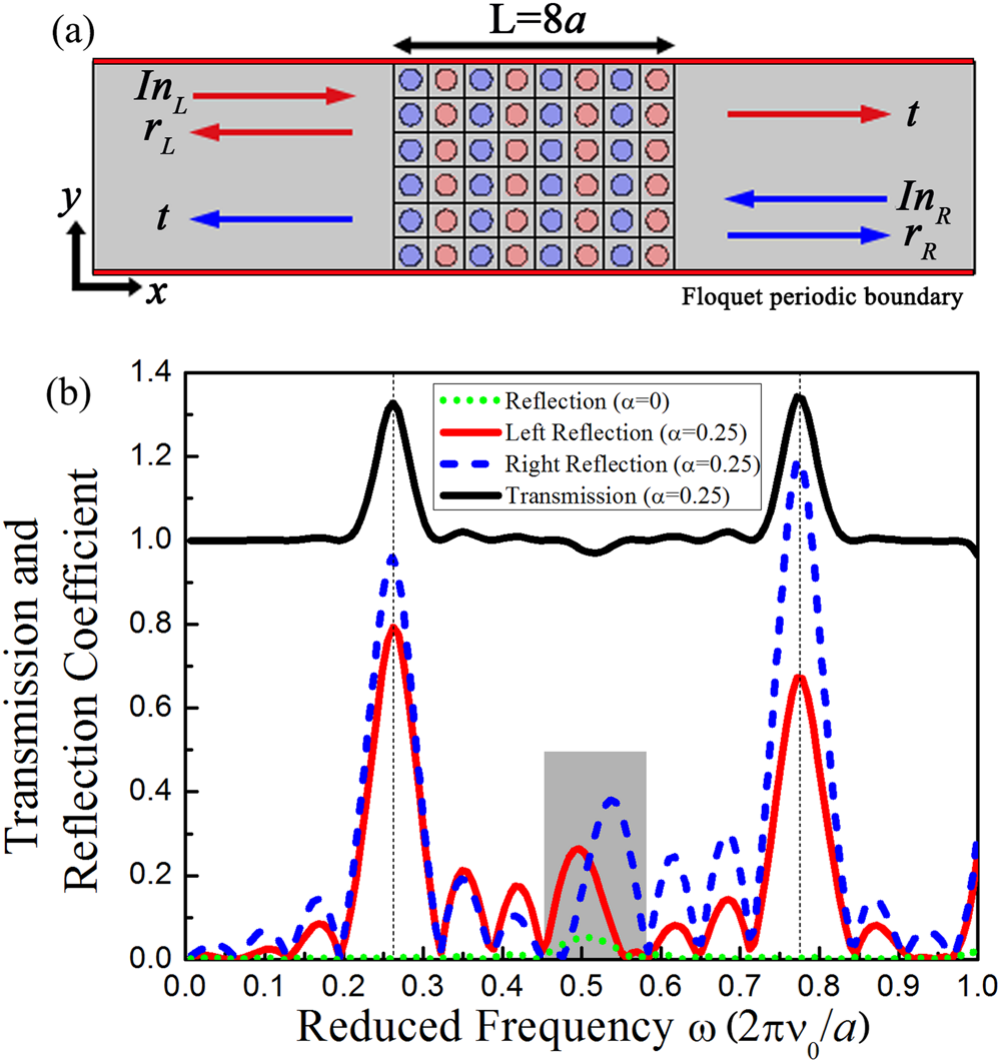
(**a**) Schematic presentation of the normal incidence model, which is composed of an 8-layer cell structure: four loss regions and four gain regions. The reflected and transmitted acoustic waves from the left/right incidence are indicated by the red/blue arrows. (**b**) Reflection coefficient (green dotted line) in the non-dissipative system and transmission (black solid line) and reflection coefficient of the left-incident (red solid line) and right-incident (blue dashed line) waves in the non-Hermitian system.

At the frequency of degenerated points, these structures exhibit a notably interesting feature of acoustic control. We use an eight-layer cell structure in the x direction, which includes four loss regions and four gain regions, as shown in Fig. 3(a). Here, the regions outside the periodic structure are consistent with the material of the background medium. Incident waves from either the left or right side are perpendicular to the structure. First, we calculate the 2D Hermitian system (α = 0). As depicted in Fig. 3(b), the reflection (green dotted line) in this system is weak because there is hardly any bandgap in the Γ-X direction in Fig. 1(a). The reflected spectrum is overlapping for the incident acoustic waves from the left or right.

In the 1D *PT*-symmetric system^33^, the acoustic waves incident from the left and right sides, possess the same transmission spectrum but different reflection spectra. Next, we have calculated the transmission and reflection coefficients of left-incident and right-incident elastic waves that propagate through the 2D *PT*-symmetric PCs. As illustrated in Fig. 3(b), the left reflection is different from the right one, and their strengths are much higher because of the gain and loss medium. High reflections and high transmissions occur at the frequencies of approximately 0.26 and 0.75, which show a great coincidence with the frequency of marked degenerated points 1 and 2 at the Γ-X direction in Fig. 1(d). The transmissions here are much higher than 1. Although the left and right reflection coefficients have different values, the overall trend is roughly identical except for the frequency region indicated in gray in which we are interested. In the marked region, two reflection peaks are distinctly interlaced, which corresponds to different unidirectional zero reflections in the frequency domain and simultaneously results in the obvious asymmetric response in this frequency range.

To better understand the unidirectional behaviors, we have focused on the gray frequency region in Fig. 3(b). The 1D *PT* system always obeys an exact “generalized unitarity relation”^27^:

1 $${r}_{L}{r}_{R}={t}^{2}(1-\frac{1}{T}),$$

where *t* is the transmission coefficient, $T\equiv {|t|}^{2}$ is the transmittance, and *r*_*L*_ and *r*_*R*_ are the left and right reflection coefficients for the incident waves, respectively.

This relation leads to another form:

2 $$|T-1|=\sqrt{{R}_{L}{R}_{R}},$$

where ${R}_{L(R)}\equiv {|{r}_{L(R)}|}^{2}$ is the reflectance. According to Eq. (1), *r*_*L*_ or *r*_*R*_ must vanish when *T* = 1. For *T* < 1, Eq. (2) can be rewritten as $T+\sqrt{{R}_{L}{R}_{R}}=1$, which is a generalization of the more familiar conservation relation *T* + *R* = 1 applied in the lossless Hermitian system, with the left and right reflectance *R*_*L*_ = *R*_*R*_ = *R*. For *T* > 1, Eq. (2) becomes $T-\sqrt{{R}_{L}{R}_{R}}=1$.

**Figure 4:**
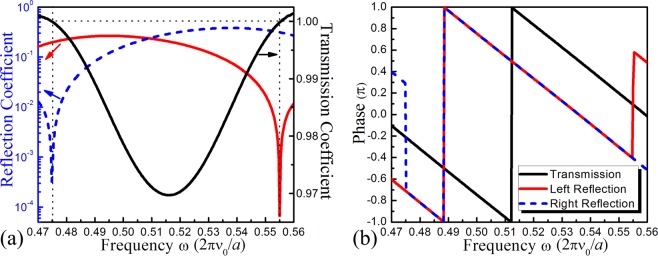
(**a**) Left (red solid line) and right (blue dashed line) reflection coefficients that correspond to the left y-axis and the transmission coefficient (black solid line) that corresponds to the right y-axis. (**b**) Phases of the left-reflected, right-reflected and transmitted waves in the asymmetric frequency range.

In our 2D *PT*-symmetric PCs, the principles applied the in 1D structure are also applicable. We discover two exceptional points (EPs) with obvious unidirectional behavior in the frequency range of our study. As shown in Fig. 4(a), the right reflection is close to 0 and the left reflection is approximately 0.20 at the frequency ${\omega }_{1}=0.475(2\pi {\nu }_{0}/a)$, whereas at the frequency ${\omega }_{2}=0.555(2\pi {\nu }_{0}/a)$, the reflections are ${r}_{L}\approx 0$ and ${r}_{R}\approx 0.32$. Figure 4(a) also shows that the transmission spectra of two incident directions are identical. A transmission valley forms because of the directional bandgap in the Γ-X direction, and two EPs are located around that bandgap. At two corresponding EPs, the transmission is exactly 1. This result presents a unidirectional behavior of reflectionless perfect transmission, where one of the reflections always remains at zero but the other is not when *T* = 1. In addition, the relation between reflection and transmission in our 2D structure excellently satisfies the generalized unitarity relation. As illustrated in Fig. 4(b), the phase difference between transmitted waves and reflected waves is always π/2 regardless of the incident direction. Moreover, the right reflected waves experience an abrupt phase transition of π at frequency *ω*_1_, while the left reflected waves experience the same transition at frequency *ω*_2_. The phase between the left and right reflections is π for the case of *T* > 1 but is invariably equal for *T* < 1. One-way transport without reflection occurs when the *PT*-symmetric phase changes into the broken phase mainly because the reflected waves are completely absorbed by the loss part. However, the unitary behavior of transmitted acoustic waves is only contributed by the gain part in the lossy system.

**Figure 5:**
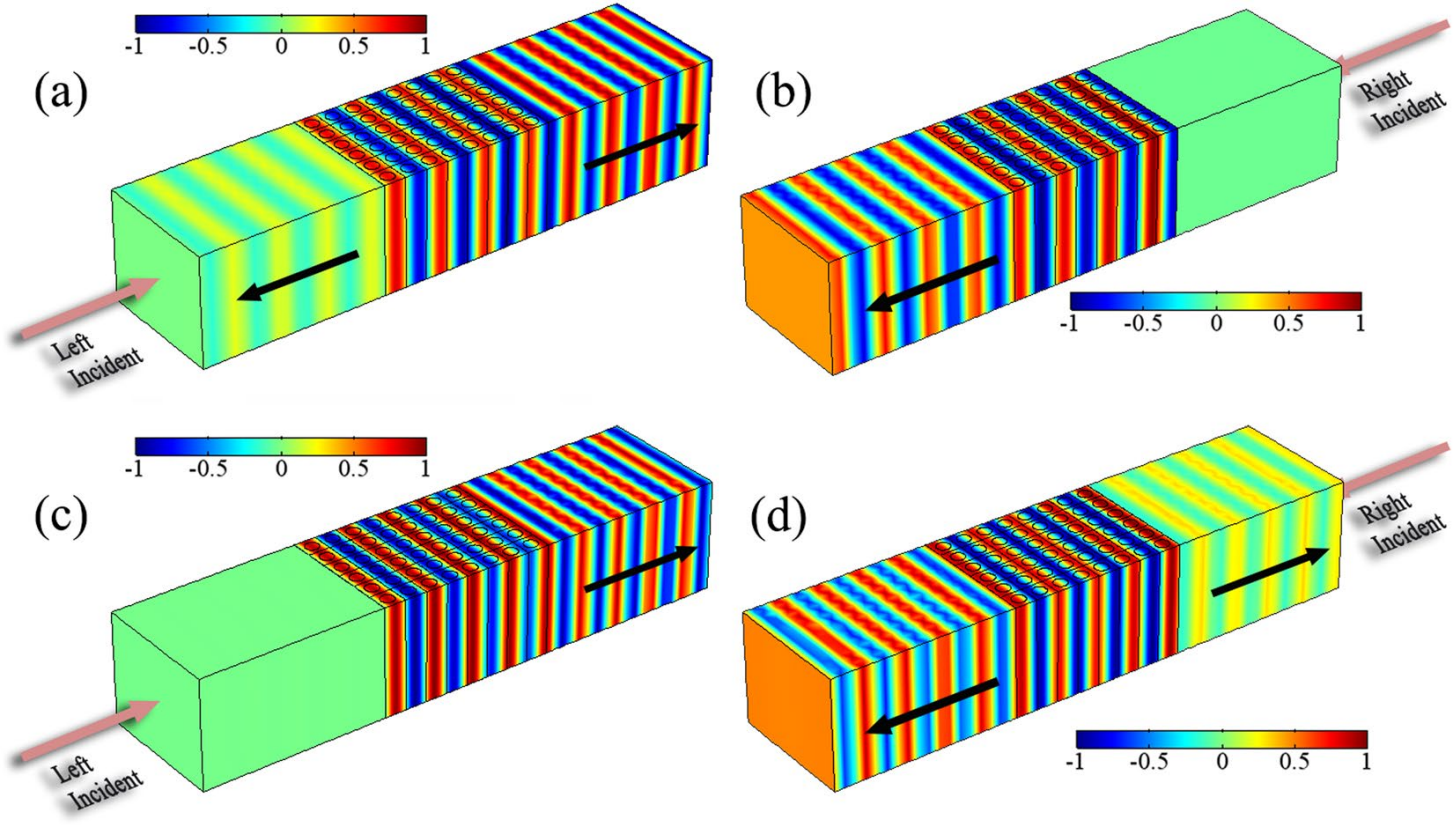
(**a,b**) Acoustic scattering pressure field for left and right incidences at *ω*_1_ = 0.475. (**c,d**) Acoustic pressure field for left and right incidences at *ω*_2_ = 0.555. Here, the amplitude is normalized. The field of incident waves is not shown and the directions of transmitted and reflected waves are indicated by black arrows.

The simulated scattering pressure fields in Fig. 5(a–d) have excellently confirmed the transmittance and reflectance results in Fig. 4(a). The incident fields are not shown. As depicted in Fig. 5(a–b), there is a significant reflection phenomenon when the waves are vertically incident at *ω*_1_ = 0.475, but the right-incident waves are reflectionless. Conversely, Fig. 5(c–d) show that the left incidence has no reflection but the right reflected wave is obviously at *ω*_2_ = 0.555. Based on the property of EPs, it can be designed into a novel acoustic switch device using the unidirectional reflectionless behavior, which is also applied to other multifunctional devices in acoustic wave manipulations.

### Acoustic flat slab focusing

**Figure 6:**
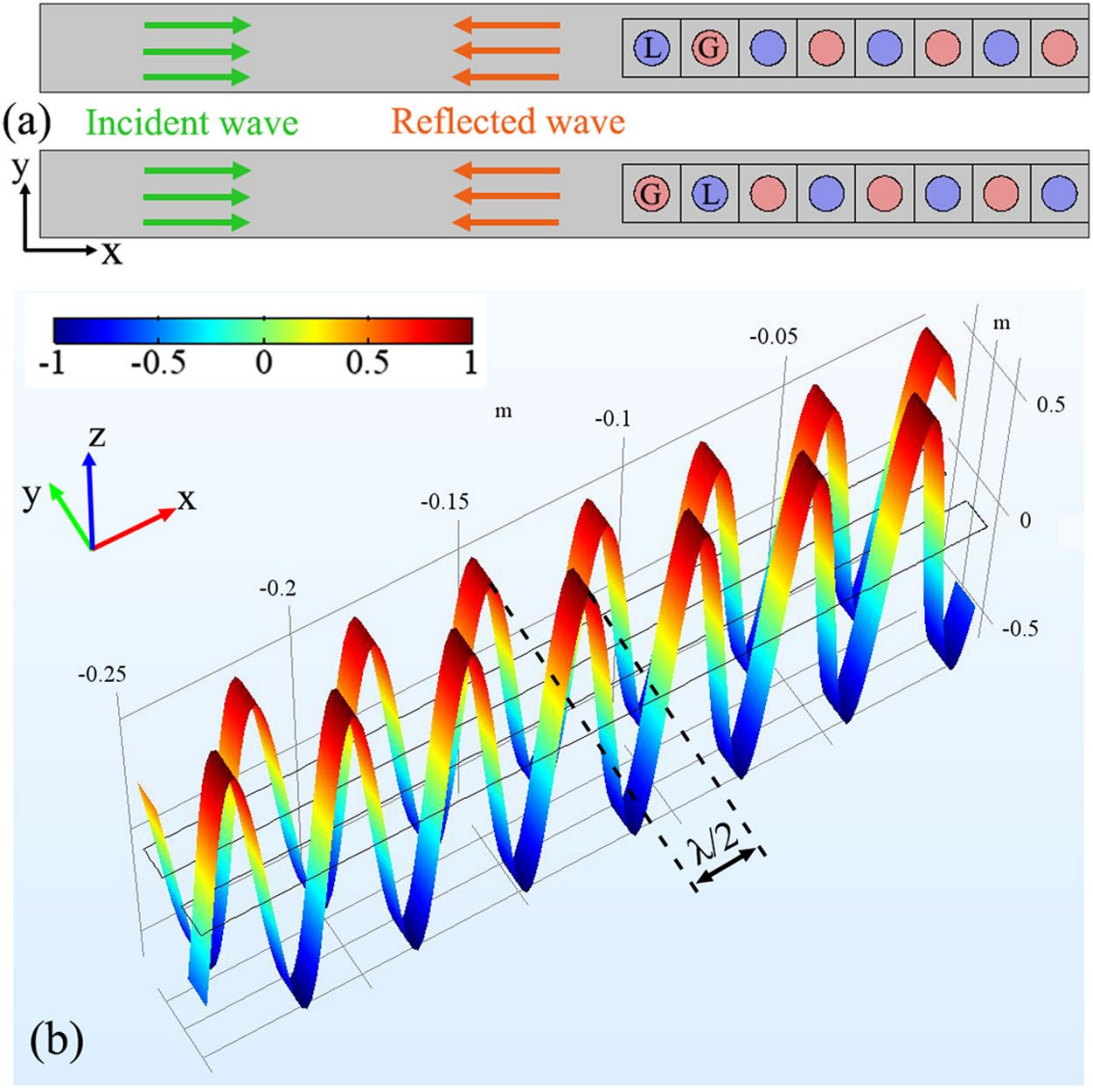
(**a**) Schematic diagram of two units for non-Hermitian metamaterials. The green and orange arrows indicate the propagation direction of incident and reflected waves, respectively. (**b**) Pressure strips of the reflected waves by the two units. The high maps of the pressure field are utilized to distinctly display the phase shift between two units. Here, the amplitude is normalized.

There is always a phase difference π between the left- and right-reflected waves when the transmittance is greater than 1. It can be utilized to frame two units of metamaterials, which can realize the discrete phase gradient of π that covers the full range of 2π. Using the above structure and parameters, Fig. 6(a) shows two units of *PT*-symmetric metamaterials. Due to different arrays of gain and loss mediums, the reflected waves of two units correspond to the left and right reflected waves, respectively. Here, the lattice length *a* is 1 cm. To clearly exhibit the discrete phase shifts, as plotted in Fig. 6(b), the pressure distribution of reflected waves at the left edge of each unit is independently calculated with free space wavelength λ = 3.5*a*. At this wavelength, the reflection amplitudes in two units are nearly equal, which are approximately 3/5 of the amplitude of the incident wave. The pressure strips are obviously shown, and there is a shift of λ/2, i.e., the π phase between adjacent peaks of two reflected waves, so that two such unit structures can shift up to provide a discrete phase gradient for the entire 2π range.

Using the 2π range phase shifts for reflected waves, we can design different metamaterials to achieve the wavefront manipulation with the combination of the generalized Snell’s law:

3 $$k[\sin ({\theta }_{r})-\,\sin ({\theta }_{i})]=\frac{1}{{n}_{i}}\frac{d\varphi }{dy},$$

where $k=2\pi /\lambda$ is the wave vector in the background medium (water), *θ*_*r*_(*θ*_*i*_) is the angle of reflection (incidence), *n*_*i*_ is the refractive indices of the media, and *dϕ*/*dy* is the phase shift per unit distance along the y direction. When the plane wave is normally incident on the metamaterials, Eq. (3) can be rewritten as:

4 $${\theta }_{r}=\arcsin (\frac{\lambda }{2\pi {n}_{i}}\frac{d\varphi }{dy}).$$

Equation (4) demonstrates that the reflected angle can be freely controlled by designing the appropriate phase profile along the y direction.

**Figure 7:**
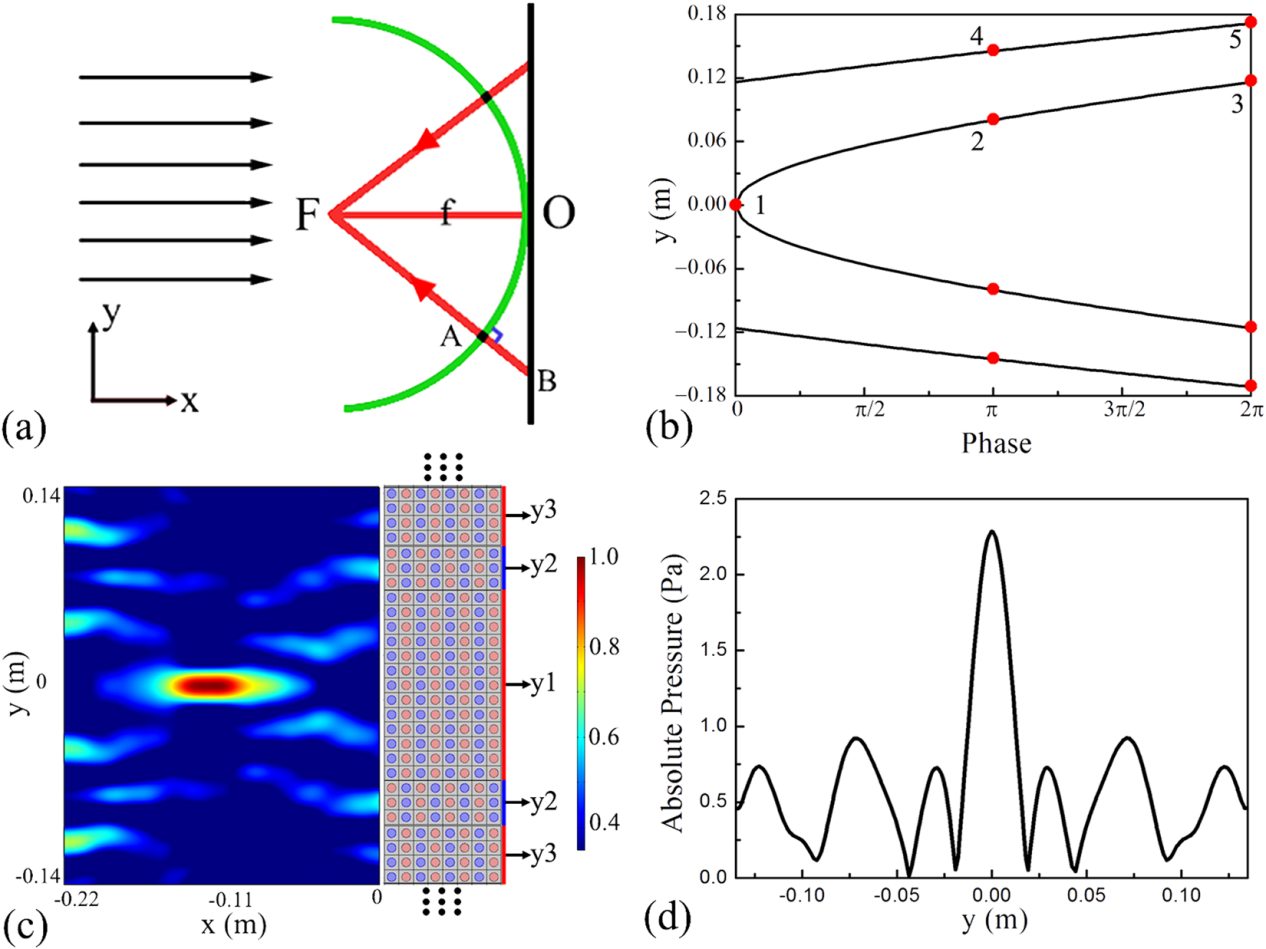
(**a**) Schematic diagram of the design of the lens. A hyperboloidal phase profile along the y direction is used to focus the acoustic plane wave to a single point at a distance *f* from the metamaterials. (**b**) Discrete phase shifts along the y direction. (**c**) Normalized absolute pressure field for the designed lens with *f* = 5λ. (**d**) Distribution of the absolute pressure in the y direction at x = −0.13 *m*.

Based on this property, we design a non-Hermitian acoustic flat lens to focus on the acoustic plane wave. Figure 7(a) shows the schematic diagram of the flat lens, which offers more possibilities for controlling the geometric freedom of metamaterials. The hyperboloidal phase profile along the y direction is used to realize acoustic point focusing at a distance *f* from the structure. For a given focal length *f*, the positions of phase shift *ϕ*(*y*) in the y direction must satisfy the equation:

5 $$\varphi (y)=k\cdot \overline{AB}=\frac{2\pi }{\lambda }(\sqrt{{y}^{2}+{f}^{2}}-f).$$

Here, we chose *f* = 5λ as the focal length. For the sake of clarity, there is a rule to convert a continuous phase profile to a discrete phase profile. Because the π phase difference of two structural arrangements, the units providing π phase shift is utilized to displace the continuous phase range π ± π/2 at the specified position in the y direction. From Eq. (5), the coordinates of discrete phase shifts are calculated to obtain y_1_ = 0, y_2_ = ±0.080 *m*, y_3_ = 0.116 *m*, and y_4_ = 0.145 *m*, as shown in Fig. 7(b). We arrange the two unit structures to construct the lens according to coordinates, and the absolute acoustic pressure field of reflected waves for the designed lens is plotted in Fig. 7(c). It is clearly observed that an excellent focusing effect occurs at the left space of the constructed lens when the plane wave is vertically incident. Figure 7(d) shows the distribution of the absolute pressure field in the vertical direction, where the coordinate of the x axis is selected as −0.13 *m* away from the outer structure. The intensity of the pressure at the focal points is approximately 2.3 times larger than that of the incident waves.

**Figure 8:**
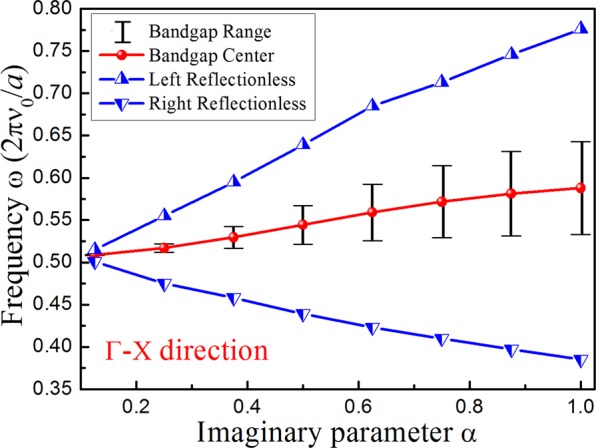
Corresponding frequency of the bandgap and left and right EPs in the Γ-X direction as a function of the gain/loss parameter α.

We have demonstrated the effect of imaginary parameter α in Fig. 8. The bandgap range and center frequency between the second and third bands in the Γ-X direction increase with the increase in α. At the bandgap frequency range, it will forms a transmission valley, so the left and right EPs (*T* = 1) are easier to find around the bandgap. On both sides of the EPs, there will always be a case of *T* > 1, which determines the frequencies of the phase difference of π. Thus, the imaginary parameter α has a great effect on the frequencies of the phase transition. We can control the frequency, which has a phase difference of π, by changing the value of the imaginary part. Theoretically, a high focusing efficiency of reflected waves can also be acquired by modulating the gain/loss strength α. Therefore, both the frequency and the amplitude of acoustic flat focusing can be manipulated.

## Discussion

We have designed a novel 2D non-Hermitian PC for acoustic flat focusing. It is composed of soft rubber rods with a complex conjugate elastic modulus, which are periodically embedded in water. When plane waves propagate through the *PT* phononic structure from opposite directions, there are significant unidirectional behaviors and phase transitions in the scattering phenomenon. The right-reflected waves vanish at ${\omega }_{1}=0.475(2\pi {\nu }_{0}/a)$, while the left-reflectionless effect appears at ${\omega }_{2}=0.555(2\pi {\nu }_{0}/a)$. Combining the generalized Snell’s law and the phase shift of two unit structures, we constructed the planar non-Hermitian metamaterial lens to achieve acoustic focusing via reflected waves. Such asymmetric response and phase modulation in our study can be used to conceive acoustic experiments and design functional devices. It also has great potential in other lattice systems, non-Hermitian metasurfaces, and even the transient time dimension.

## Methods

### Numerical simulation of the structures

The 2D *PT*-symmetric PC considered in this paper is composed of gain/loss materials in water (${\rho }_{0}=1000\,kg/{m}^{3}$, ${\nu }_{0}=1490\,m/s$). Both the gain and loss materials are soft rubber (${\rho }_{L(G)}=950\,kg/{m}^{3}$), but their longitudinal wave velocities are distinguished by positive and negative imaginary parts (${\nu }_{L(G)}=1550\times (1\pm \alpha i)m/s$). To simplify the calculation process in our case, we ignore the shear modulus in rubber because it does not significantly affect the essential physics of the system. The z direction in this *PT* system is infinitely long, and the acoustic waves propagate along the x direction. Throughout this paper, the finite-element method (FEM) based on the software COMSOL Multiphysics 5.3a is employed for the simulations. The calculations of the band structure are performed with the super cell method. For Fig. 3(a), the background pressure field of plane waves is used, and periodic boundary conditions are imposed to the upper and lower boundaries to simplify the structure and reduce the amount of calculation without losing accuracy. Each strip of the pressure pattern in Fig. 6(b) is independently calculated. Perfectly matched layers (PMLs) are also used in Fig. 7(c) to eliminate the reflected waves by the outer boundaries.

### Realization of the structures

The structures consist of the loss and gain medium with a positive and negative imaginary part respectively. Generally, the acoustic loss materials are ubiquitous in nature because acoustic loss can be easily caused by viscous elasticity. It should be noted that a natural acoustic gain media has not yet been found. However, it can be effectively realized by well-designed feedback systems^39^. More specifically, the proposed structures can be realized by using the sensors and pre-amplifiers. The sensed signal is amplified in a pre-amplifier of appropriate gain progression. In addition, an equivalent acoustic gain system can be constructed by combining auxiliary devices such as microphones and loudspeakers.
